# Inhibition of sepsis-induced organ injury

**Published:** 2013-07

Sepsis is a complex condition in which a systematic inflammatory response is activated by infection, setting off a cascade of events that can culminate in multiple organ failure and death. New therapeutic strategies are urgently needed, with a key goal being the development of drugs that can be administered when sepsis is already underway. Inhibition of the transcription factor NF-κB has been shown to have a protective role in sepsis. Here, Christoph Thiemermann and colleagues tested the effect of inhibiting the IKK complex, which is involved in the early stages of NF-κB activation, on organ dysfunction and injury in mouse models of sepsis. They report that sepsis-induced organ injury is attenuated upon delayed administration of an IKK inhibitor, suggesting that this inhibitor could be given to sepsis patients during the devastating late stages in disease progression. **Page 1031**

